# A cross-sectional study of the relationship between depression status, health care coverage, and sexual orientation

**DOI:** 10.1007/s44192-023-00039-0

**Published:** 2023-06-29

**Authors:** Yang Liu, Megan A. O’Grady

**Affiliations:** Department of Public Health Sciences, School of Medicine, University of Connecticut, Farmington, USA

**Keywords:** Health care coverage, Depression, Sexual minority, Mental health

## Abstract

Health care coverage is an important factor in receipt of behavioral healthcare. This study uses data from the New York City Community Health Survey to examine how sexual minority status impacts the relationship between depression status and having health care coverage. Approximately 10% of the sample (n = 9571; 47% 45+ years old; 35% White Non-Hispanic; 7% sexual minority) reported probable depression and low health care coverage. Compared to heterosexual participants, a greater proportion of sexual minority participants had low health care coverage (17% vs. 9%) and probable depression (19% vs. 9%). Logistic regression examining the association between probable depression status and health care coverage showed that those with probable depression have odds of low health care coverage that are were 3.08 times those who did not have probable depression; this relationship was not modified by sexual orientation. Continued research to understand the interplay of health care coverage, mental health, and sexual orientation is needed.

## Introduction

### Background

Depression is a significant worldwide health issue that may have been further exacerbated by the COVID pandemic [38]. In the United States (US), well before the COVID-19 pandemic, depression was already a growing health issue and becoming more prevalent [21]. According to the Substance Abuse and Mental Health Services Administration (SAMHSA)’s National Survey on Drug Use and Health (NSDUH), an estimated 21 million adults in the US have experienced at least one major depressive episode (MDE) and that number represented 8.4% of the US adult population in 2020 [15, 29]. Depression can have severe impacts on individual and public health. For example, studies suggested that depression is strongly related to other chronic diseases and deteriorated quality of life [4, 5, 26, 41].

While there are a variety of evidence-based treatment options for depression, many individuals do not receive them. For example, among adults who reported a MDE in the past year, 44% did not receive treatment in the past year and one-third of people with any mental illness believe there is an unmet mental health treatment need [37]. The most common reason for not receiving mental health services in the past year among adults who had a mental illness but did not receive services was that they could not afford the cost of care [37]. One major reason that people could not afford the cost may be lack of insurance coverage [45]. Research has found that 72% of adults with mental illnesses reported at least one structural barrier to accessing treatment (e.g., high cost, low knowledge about resources) and uninsured individuals reported significantly more structural barriers than insured individuals [45]. Therefore, it is imperative to further investigate factors associated with ability to afford services (e.g., insurance status, unaffordable medical cost) among those with depression in order to improve access to treatment and close gaps in unmet treatment need.

#### Mental health among sexual minority populations

Mental illness symptoms tend to be more severe among sexual minority populations. In 2019, according to the NSDUH, two in five lesbian, gay, and bisexual (LGB) adults had a serious mental illness [36]. Another study found 26% of sexual minority adults engaged in mental health services in the past year, compared to only 14% of heterosexual adults, indicating a greater treatment need among sexual minority populations [9]. Thirteen percent of LGB adults who have experienced severe mental illness symptoms tend to experience interference with their daily life functioning, compared to only 4% among heterosexual adults who experienced the same problem [22].

There was a significant increase in having major depressive disorder (MDD) among LGB young adults in 2019 (estimated to be 1.5 million or 33% of the same LGB populations in the same year) compared to 2016 where the prevalence of MDD was around 26% [36]. When compared to the heterosexual population, sexual minority young adults reported significantly more depressive symptoms in both men and women [18]. Therefore, given the greater risk of mental illness symptoms among sexual minority adults, they are important populations on which to focus investigations of depression and health care coverage status.

#### Health care coverage among sexual minority populations

There are significant disparities in terms of health care coverage between sexual minority populations and heterosexual populations. Previous research has found LGB populations were less likely to have health insurance coverage compared to their heterosexual counterparts [3, 7]. Despite suggestions that the implementation of Affordable Care Act (ACA) and legalization of same-sex marriage might have reduced the insurance coverage gap, one study still found that LGB adults were significantly more likely than heterosexual adults to report having avoided necessary medical care because of cost and had nearly twice as many bad mental health days [28].

#### Theoretical framework

**Fig. 1:**
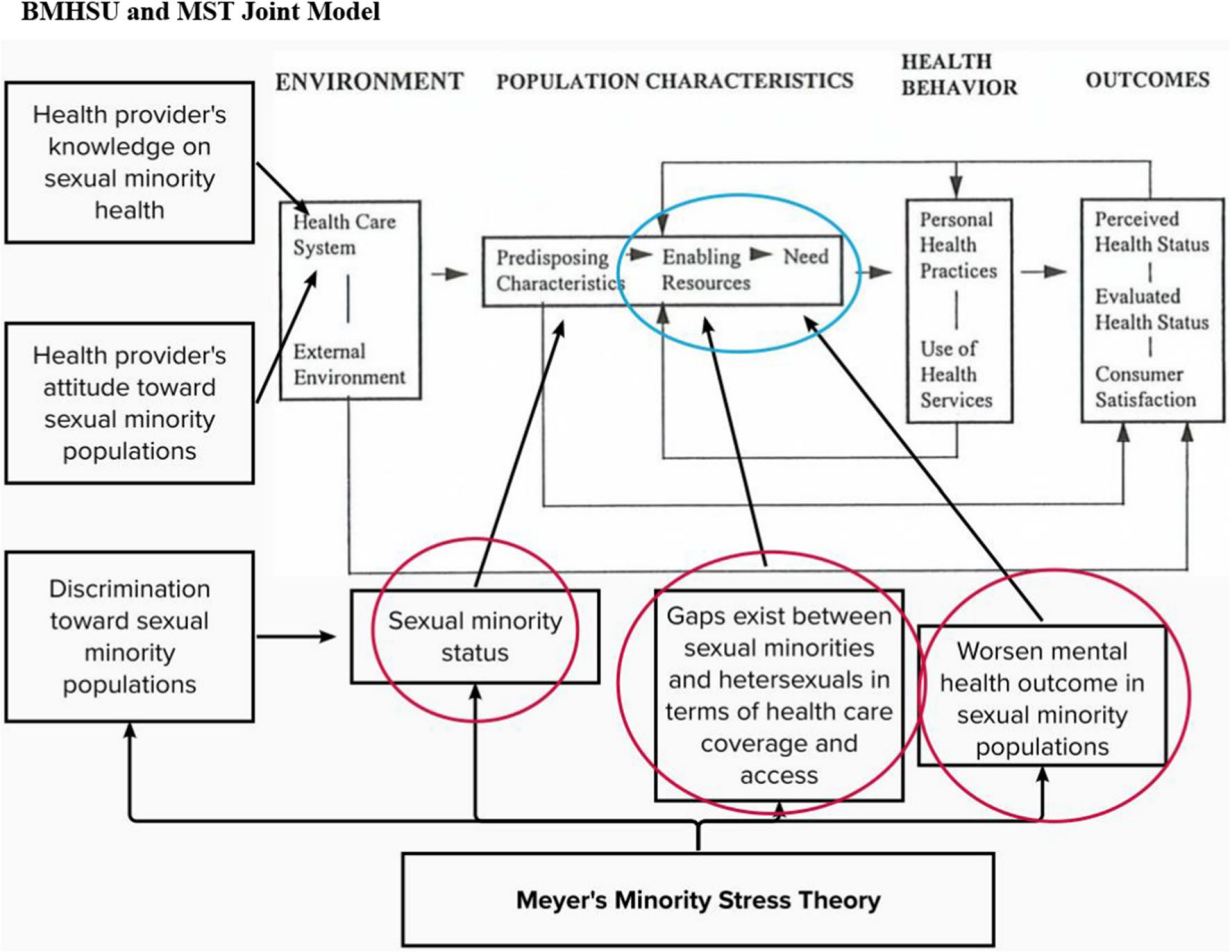
BMHSU and MST joint model

Meyer’s *Minority Stress Theory* (MST) and Andersen’s *Behavioral Model of Health Service Use* (BMHSU) serve as frameworks for this study. The MST describes mental health outcomes in minority populations and Andersen’s model describes factors affecting access and utilization of healthcare services [19, 25, 34]. According to the MST, minority status often interacts with other factors which creates synergistic effect that put minority populations at greater risk of mental illnesses [24]. While the lack of health care coverage affects all individuals’ mental wellbeing, minority populations such as sexual minorities are at greater risk of mental illnesses compared to the heterosexual population due to lack of coverage or lack of professional knowledge, skills, and cultural competencies from the health professionals to provide quality LGBT care [12, 35, 39]. Meanwhile, in Andersen’s BMHSU, three main categories of population characteristics are proposed that influence health behavior and outcomes. These include (a) pre-disposing characteristics such as social structural and demographic factors (e.g., sexual minority status), (b) enabling factors, including health insurance status and ability to pay for services, and (c) need indicators such as illnesses or conditions requiring health services (e.g., depression). While Andersen’s model does not specifically focus on mental health services, a study using BMHSU found those with the most mental health needs (e.g., people with depression) tend to be most likely to seek mental health services, which often requires some form of health care coverage in order to access [10]. It becomes a vicious cycle when sexual minorities with depression cannot access mental health service due to lack of proper insurance coverage or ability to pay, potentially further contributing to depression symptoms. In Fig. 1, we outline how the theoretical models may jointly influence healthcare coverage among those with depression who are sexual minorities. We highlight the areas of the model in which this study will focus on. For example, in Andersen’s model we focus on pre-disposing, enabling, and need factors and indicate how the MST could explain each component. In order to close the gap in access to depression care, disparities in health care coverage between sexual minority and heterosexual populations should be investigated. This may help identify policy and practice interventions to improve engagement in treatment and access to health insurance coverage.

#### Research aims

Guided by the theoretical models described above, the current study aimed to examine the relationship between probable depression status, health care coverage status, and sexual orientation status. The research aims include examining (1) the general association between probable depression and health care coverage and (2) the joint effect of probable depression and sexual minority status on the odds of not having proper health care coverage to determine whether there is a significant difference in low health care coverage outcomes between sexual minorities and heterosexual populations in a population-based study of adult New York City residents. The hypotheses are: (1) Among the general population, people with probable depression would have greater odds of having low health care coverage than those without probable depression and (2) sexual orientation will serve as a modifier; such that people with probable depression within sexual minority populations would have greater odds of having low health care coverage compared to the heterosexual population with probable depression.

## Methods

### Data source and study design

Publicly available data from the 2018 New York City Community Health Survey (NYC-CHS) were used to conduct this study. The NYC-CHS is a collection of cross-sectional, population-based telephone surveys with an annual sample of approximately 10,000 randomly selected adults aged 18 and older from all five boroughs of New York City. The Community Health Survey (CHS) has been conducted annually by the New York City Department of Health and Mental Hygiene since 2002 [27]. The data provided important information to better understand the health and risk behaviors of New York residents [27]. The rationale behind choosing 2018 dataset is to avoid any impact on the study results that may have occurred during newer data collection that occurred during the COVID-19 pandemic.

### Participants and procedures

The target population of the CHS includes adults in non-group quarters aged 18 and older who live in New York City and have a cellular telephone or live in a household with a landline telephone [27]. Prior to 2009, the CHS only included those living in households with a landline telephone [27]. The NYC-CHS uses a stratified random sample to produce neighborhood and citywide estimates [27]. Neighborhoods are defined using the United Hospital Fund’s (UHF) neighborhood designation, which assigns neighborhood based on the ZIP code of the respondent [27]. A computer-assisted telephone interviewing (CATI) system was used to collect survey data from selected respondents (n = 10,076) with landline telephones and/or cell phones (New York City [27]. The analytic sample for this study comprised participants who reported their age and sexual orientation (n = 9571).

### Measures

#### Depression symptoms

The independent variable of the study was probable depression status. Symptoms of depression were measured with the 8-item Patient Health Questionnaire (PHQ-8). The NYC Department of Health and Mental Hygiene have been using the PHQ-8 to measure any form of mental illness since 2016 CHS. The PHQ-8 score is a sum of 8 items that ask frequency of experiencing Diagnostic and Statistical Manual of Mental Disorders (DSM-5) symptoms of a major depressive episode in the past 2 weeks (not at all, several days, more than half the days, and nearly every day). The score range is from 0 to 24, with greater scores indicating greater symptom frequency, and probable depression is noted for scores greater than or equal to 10 [17]. The PHQ-8 depression scale has been frequently used to assess depression in general population studies and has demonstrated good validity and reliability [17, 40]. The PHQ-8 showed excellent internal consistency reliability in the current study sample (Cronbach’s alpha = 0.8436).

#### Sexual orientation

Sexual orientation was categorized into two groups (heterosexual vs. sexual minority; included lesbian/gay/bisexual/other sexual orientation).

#### Study outcome: healthcare coverage

The outcome of the study was health care coverage, specifically related to one’s health insurance status and ability to cover medical costs. It was defined by combining insurance status (insured vs. uninsured) and whether the participants had any difficulty to cover out-of-pocket medical costs in the past 12 months (yes/no). Participants were divided into two groups: (1) those who were insured and had not experienced unaffordable medical costs (“high coverage”) and (2) those who were either insured or uninsured but had experienced unaffordable medical costs (“low coverage”). The rationale behind combining these two variables was that health insurance status alone rarely explains its association with mental illness, and previous research found that difficulty of paying copayment did exist among insured LGB populations [12].

#### Covariates

Important sociodemographic and health factors are included similar to other studies on mental health among sexual minorities [2, 9, 14, 16, 20]. Sociodemographic variables included age, sex at birth (male/female), race, marital status, educational attainment, and employment status (employed/not employed). Age was categorized into three groups (18–24 years, 25–44 years, 45+). Race/ethnicity was categorized into three groups (White non-Hispanic, Black/other non-Hispanic, and Hispanic; Asian/Pacific Islander non-Hispanic and other non-Hispanic were combined with the Black non-Hispanic population to accommodate for small sample sizes). Education included four levels (less than high school, high school graduate, some college, and college graduate). Participants were also asked to rate their health on a scale that consisted of five responses (excellent, very good, good, fair, and poor). Self-rated health was categorized into two levels (excellent/very good/good and fair/poor) Marital status was categorized into two groups: married and not married. Participants were also asked whether they have someone they consider as their primary care physician or personal doctor (yes/no) and if there was a time in the past 12 months when they needed medical care but did not get it (yes/no).

### Statistical analyses

All analyses were conducted using STATA/SE version 17 [43], and procedures were used to account for the complex survey design and weighting of NYC-CHS. Weighted data is presented in Tables 1 and 2. Sociodemographic and other descriptive information about the study population is included using the tabulate procedure. Table 1 shows whether there are significant differences in demographic characteristics between heterosexual and sexual minority populations based on chi-square analysis. To test for hypothesis (1), the main association between health care coverage and depression are examined by conducting logistic regression, both crude and after adjusting for covariates. Crude odds ratios (OR) and adjusted odds ratios (aOR), along with their corresponding p-value and 95% confidence intervals (CI), were reported. To test hypothesis (2), an interaction term between probable depression and sexual orientation was included in adjusted models to test for their statistical interaction. Statistical significance was set at p < 0.05.

**Table 1:** Health care coverage and probable depression characteristics, overall and by sexual orientation, New York City Community Health Survey (NYC-CHS), 2018 (n = 9571)

Sample characteristics (n = 9571)	N (weighted %)	N (weighted %)	N (weighted %)	P-value
	Overall	Gay/lesbian/bisexual/other n = 634 (7.28)	Heterosexual n = 8937 (92.72)	
Birth sex				0.0492
Men	4156 (46.65)	343 (53.34)	3813 (46.20)	
Women	5380 (53.35)	288 (47.66)	5092 (53.80)	
Age groups				< 0.001
18–24	690 (13.00)	76 (20.47)	614 (12.41)	
25–44	2910 (40.32)	215 (47.86)	2695 (39.73)	
45+	5955 (46.68)	343 (31.66)	5612 (47.86)	
Race				0.0018
White, non-Hispanic	3484 (35.40)	251 (39.59)	3124 (35.90)	
Black/others, non-Hispanic	3697 (37.62)	169 (28.66)	3391 (39.01)	
Hispanic	2895 (26.97)	214 (31.74)	2422 (25.09)	
Marital status				< 0.001
Married	3786 (41.86)	137 (18.96)	3469 (43.40)	
Not married	6190 (58.14)	492 (81.04)	5392 (56.60)	
Country of origin				< 0.001
US born	5601 (53.49)	451 (69.57)	4993 (53.73)	
Foreign born	4413 (46.51)	181 (30.43)	3898 (46.27)	
Employment status				0.119
Employed	5404 (60.08)	392 (64.90)	4781 (60.13)	
Not employed	4604 (39.92)	239 (35.10)	4105 (39.87)	
Education				0.0008
Less than high school	1555 (18.18)	74 (11.60)	1277 (16.79)	
High school graduate	2148 (24.19)	96 (17.47)	1949 (24.65)	
Some college	2127 (22.67)	129 (25.79)	1931 (23.19)	
College graduate	4176 (34.97)	331 (45.14)	3728 (35.37)	
Self-rated health				0.1534
Excellent/very good/good	7368 (77.10)	498 (81.25)	6590 (77.72)	
Fair/poor	2662 (22.90)	135 (18.75)	2316 (22.28)	
Didn’t received needed care				0.0144
Yes	1062 (11.20)	92 (15.20)	881 (10.43)	
No	8951 (88.80)	538 (84.80)	8010 (89.57)	
Presence of primary care provider				0.012
Yes	8755 (84.28)	546 (79.41)	7824 (85.34)	
No	1256 (15.72)	82 (20.59)	1060 (14.66)	
Health care coverage				0.0001
Low coverage	824 (9.76)	74 (16.99)	707 (9.01)	
High coverage	8061 (90.24)	498 (83.01)	7237 (90.99)	
Probable depression				< 0.001
Yes	955 (10.22)	95 (19.08)	802 (9.33)	
No	8449 (89.78)	496 (80.92)	7594 (90.67)	

Frequencies may not add up to total sample frequencies due to exclusion of missing values, “refused”, and “don’t know” responses. P-values indicate whether there is a significant different in demographic characteristics between heterosexual and sexual minority populations

**Table 2:** Characteristics of those with low health care coverage: depression status and sexual orientation, New York City Community Health Survey, 2018 (n = 770)

Sample characteristics (n = 770)	N (%)	N (weighted %)	N (weighted %)	P-value
	Overall	Gay/lesbian/bisexual/other n = 68	Heterosexual n = 667	
Probable depression				0.3544
Yes	181 (24.78)	20 (31.40)	152 (23.98)	
No	589 (75.22)	48 (68.60)	515 (76.02)	

Frequencies may not add up to total sample frequencies due to exclusion of missing values, “refused”, and “don’t know” responses. P-value presented represents the results of chi-square analysis examining the difference in depression status between heterosexual and sexual minority populations among the low health care coverage group)

## Results

### Sample description

The sample is described in Table 1. Slightly more than half of the sample were women (53.35%). About 93% of the sample reported their sexual orientation as heterosexual and 7% as one of the sexual minority groups. Within the sexual minority group, there were more men than women (53.34% and 47.66%, respectively). About half of the study sample were older than 45 years (46.68%). Overall, 7735 (90.40%) individuals reported they were insured and had no troubles to pay the out-of-pocket cost (high health care coverage) and 781 (9.60%) individuals reported they were either insured or uninsured but had difficulty paying out-of-pocket medical costs even though they were insured (low health care coverage) (Table 1). There were 955 individuals that reported probable depression which accounts for roughly 10% of the sample (Table 1). Compared to heterosexual participants, a greater proportion of participants in the sexual minority group had low health care coverage (16.99% vs. 9.01%, p = 0.0001) and met criteria for probable depression (19.08% vs. 9.33%, p < 0.001) (Table 1).

Table 2 illustrates depression status and sexual minority status among the low healthcare coverage group (n = 770). In this low healthcare coverage group, the proportion of people with probable depression in the sexual minority group as compared to the heterosexual group (31.4% vs. 23.98%) was not significantly different.

#### **Hypothesis 1**

Do people with probable depression have greater odds of having low health care coverage than those without probable depression?

After controlling for covariates, there was a significant difference in the odds of having low health care coverage between those with probable depression compared to those without probable depression (aOR: 3.08; 95% CI 2.16, 4.40) (Table 3: Model 1).

**Table 3:** Association between low health care coverage, probable depression, and sexual orientation, New York City Community Health Survey (NYC-CHS), 2018 (n = 9571)

	Crude OR (95% CI)	Adjusted OR (95% CI)^a^
Probable depression (Model 1)		
No	Ref.	Ref.
Yes	3.37 (2.56, 4.44)**	3.08 (2.16, 4.40)**
Sexual orientation moderation (Model 2)		
Sexual orientation * probable depression		
Probable depression	3.61 (2.68, 4.87)**	3.04 (2.11, 4.40)**
Sexual orientation (gay/lesbian/bisexual/other)	2.16 (1.38, 3.37)*	1.74 (1.06. 2.86)*
Sexual orientation (gay/lesbian/bisexual/other) * probable depression	0.61 (0.25, 1.48)	1.09 (0.39, 3.02)

*OR* odds ratio, *CI* confidence interval

*p < 0.05; **p < 0.001

^a^Adjusted for age, sex, race, marital status, country of origin, employment status, education level, self-rated health, didn’t receive needed care, presence of primary care physician

#### **Hypothesis 2**

Does sexual orientation modify the relationship between probable depression and healthcare coverage?

In the adjusted model examining whether sexual minority status moderates the relationship between depression status and low healthcare coverage, the interaction term was not significant (aOR: 1.09; 95% CI 0.39, 3.02) (Table 3: Model 2); therefore, sexual minority status does not modify the relationship between probable depression and health care coverage in this study.

## Discussion

Depression is a prevalent condition in the US and worldwide and constitutes a major public health issue. Unfortunately, access to depression care can be impeded by various factors (e.g., inability to pay). It is important to understand factors related to ability to pay for services in order to develop targeted policies to improve coverage gaps. In this study, we examined whether those with probable depression were more likely to have low health care coverage than those without probable depression and whether sexual orientation modifies this relationship. The prevalence of probable depression in this sample was higher than the national prevalence that was reported in 2017 (10.22% vs. 7.10%) but was lower than the lifetime prevalence of depression in previous research (10.22% vs. 16.20%) [15]. Results suggest that depression status may be a key indicator of ability to pay for healthcare coverage.

Our first hypothesis, that those with depression would have lower health care coverage, was supported. These findings were consistent with some previous studies [8, 11]. For example, a previous study found that people diagnosed with depression had significantly higher annual health care costs and higher costs for every category of care (e.g., primary care, medical specialty, medical inpatient, pharmacy, laboratory) [42]. This could be a potential explanation for why lower health care coverage was observed among people with probable depression since greater medical utilization exceeds direct treatment costs for depression [42]. This explanation is also somewhat coherent with perspective from the BMHSU due to previous research finding those with the most mental health needs (e.g., people with depression) tend to be most likely to seek mental health services [10]. These mental health services are usually expensive in terms of cost so insurance companies might be reluctant to provide coverage.

Our second hypothesis was not supported; the relationship between depression and health care coverage was not modified by sexual orientation. This finding is contrary to some previous studies where sexual orientation was found having major effect on healthcare access and significant disparities in access to mental health care was observed between sexual minorities and heterosexual populations [44, 46]. The lack of significant moderation in this study can be potentially explained by two rationales: (1) the small sample size of sexual minority populations in this study made the moderation relatively undetectable and (2) probable depression was a robust predictor on its own toward predicting low health coverage, which could potentially mask the effect of sexual orientation. Nonetheless, sexual minorities still suffer from disproportionate mental health burden and health care coverage gaps according to the previous studies and the results from this study [2, 12, 22, 23, 33]. According to the BMHSU model, health care coverage is an enabling factor for addressing health care needs. On the positive side, we found that the majority of participants reported high healthcare coverage (90%). Unfortunately, we found that sexual minority groups were significantly more likely to have low healthcare coverage than the heterosexual group (17% vs. 9%, respectively). This identifies a health care coverage gap between sexual minority populations and the heterosexual population that may contribute to poorer healthcare outcomes among sexual minorities. Future research should be conducted to understand factors that lead to this gap and ways to mitigate it.

Despite our finding that the relationship between probable depression and health care coverage was not modified by sexual orientation, higher prevalence of probable depression was observed among sexual minorities. This observation is consistent with the MST’s perspective where negative mental health outcomes were more likely to occur among people with minority status [18, 20, 24, 25]. Possible explanations for high prevalence of probable depression among sexual minorities were lack of access to services and social support [6, 8]. The reported disparities in probable depression between sexual minority populations and the heterosexual group highlights the need for increasing outreach and tailored services for sexual minority groups. There are already existing interventions that aim to reduce minority mental stress such as project ESTEEM (Effective Skills to Empower Effective Men) and EQuIP (Empowering Queer Identities in Psychotherapy) [30, 31]. Additional implementation of interventions similar to ESTEEM or EQuIP are needed to efficiently address the unmet mental health needs in sexual minority populations. The results from this study highlight the evidence that the gap in mental health and health care coverage among sexual minority populations is large and should be urgently addressed.

### Limitations and bias

There are some limitations to consider. First, the NYC-CHS were collected through phone interview thus all responses were self-reported. Certain information might be selectively revealed or suppressed by participants due to social-desirable responses. Secondly, the small sample size of the sexual minority populations could limit the precision of estimates. The effect of the interaction between probable depression status and sexual minority status might have been masked due to this small sample size. Thirdly, the results from this study may not generalizable outside of NYC. Lastly, the NYC-CHS data were collected before the onset of COVID-19 pandemic, thus the study results could not represent the current mental health and health care coverage situation. However, based on the evidence from recent studies [1, 13, 32, 38], mental health and mental health utilization are assumed to have worsened since the COVID-19 pandemic. However, there are also strengths, including combining two highly utilized theories to guide the work. To our knowledge, Meyer’s *Minority Stress Theory* and Andersen’s *Behavioral Model of Health Service Use* had not been previously combined to examine the interaction effect of sexual minority status and probable depression on health care coverage. Further, the study uses a representative sample from a longstanding survey of New York City adults using complex sampling methods.

### Conclusions and future research

The finding from this study suggested that disparities in health care coverage due to disparities in probable depression occurs in the general population but no significant difference was observed between sexual minority populations and heterosexuals. Utilizing a larger sample size of sexual minority populations is recommended in future studies. In addition, it is possible that additional analyses may uncover a relationship among the variables we examined. For example, it is plausible that healthcare coverage may modify the relationship between minority status and depression, such that individuals with coverage might be less likely to experience depression. Furthermore, besides health care coverage outcome, future research can also shift to examine the perceived health outcome(s) in populations such as racial minorities, gender minorities, and those with a lower educational degree as probable depression was prevalent in these populations. Perceived health discrimination is a particular outcome that might be worth of examination in relation to sexual and racial minority status. Also, since NYC-CHS only included adult participants, future research that focuses on youth populations is also recommended. Lastly, this study could serve as a reference study for similar future research that is done in a post-COVID-19 timeframe to observe the effect of how COVID-19 impact mental health and health care coverage across different populations.

## Data Availability

The data came from the publicly available 2018 New York City Community Health Survey (NYC-CHS) dataset. Code used for analysis is available in STATA.
